# Voids and marginal gaps in oval-shaped root canals filled with calcium silicate-based sealers: Micro-CT study

**DOI:** 10.1590/0103-644020256732

**Published:** 2026-01-12

**Authors:** Isabella Marian Lena, Cristiana Pereira Malta, Tamyres Veleda Fonseca, João Paulo Mendes Tribst, Amanda Maria de Oliveira Dal Piva, Cornelis Johannes Kleverlaan, János Kodolányi, Gabriel Kalil Rocha Pereira, Patrícia Maria Poli Kopper Móra, Renata Dornelles Morgental

**Affiliations:** 1 Programa de Pós-Graduação em Ciências Odontológicas, Universidade Federal de Santa Maria(UFSM), Santa Maria, Rio Grande do Sul, Brasil; 2 Programa de Pós-Graduação em Odontologia, Universidade Federal do Rio Grande do Sul(UFRGS), Porto Alegre, Rio Grande do Sul, Brasil; 3 Department of Reconstructive Oral Care, Academic Centre for Dentistry Amsterdam (ACTA), Universiteit van Amsterdam and Vrije Universiteit, Amsterdam, North Holland, The Netherlands; 4 Department of Dental Materials Science, Academic Centre for Dentistry Amsterdam (ACTA), Universiteit van Amsterdam and Vrije Universiteit, Amsterdam, North Holland, The Netherlands

**Keywords:** Bio-C Sealer, BioRoot RCS, calcium silicate-based sealers, micro-CT

## Abstract

This study investigated the percentage of voids and marginal gaps in oval root canals filled with pre-mixed or powder-liquid calcium silicate-based sealers, using the single-cone technique. Eighty-four mandibular anterior teeth with oval root canals were selected and prepared by Reciproc R25 instruments. Teeth were then divided into two groups based on the sealer: Pre-mixed (BCS, Bio-C Sealer) (n=42) or Manipulated (BRR, BioRoot RCS) (n=42). Microcomputed tomography (micro-CT) was used to evaluate the percentage of voids and marginal gaps in the apical, middle, and cervical thirds of the root canals. Surface morphology of each sealer was evaluated on bovine dentin discs by scanning electron microscopy (SEM). Mann-Whitney and Friedman tests were employed for statistical analysis (α=5). BCS presented lower percentages of marginal gaps (p=0.00) and overall empty spaces (voids + gaps, p=0.01) compared to BRR. While BRR exhibited the lowest percentage of marginal gaps in the cervical third (p=0.00), BCS showed consistent performance across all root thirds (p>0.05). SEM showed that BCS has a homogeneous structure and compact structure, while BRR exhibits a more porous surface with a greater number of mostly micron-sized or larger voids. BCS exhibited superior filling ability in oval root canals compared to BRR. The BRR group showed the highest percentage of gaps in the middle and apical segments.

## Resumo

O presente estudo investigou a porcentagem de *voids* e *gaps* em canais radiculares ovais obturados com cimentos à base de silicato de cálcio pré-misturados ou em pó-líquido, utilizando a técnica de cone único. Oitenta e quatro dentes anteriores inferiores com canais radiculares ovais foram selecionados e preparados com instrumentos Reciproc R25. Os dentes foram divididos em dois grupos conforme o cimento utilizado: pré-misturado (BCS, Bio-C Sealer) (n=42) ou manipulado (BRR, BioRoot RCS) (n=42). Microtomografia computadorizada (micro-CT) foi utilizada para avaliar a porcentagem de *voids* e *gaps* nos terços apical, médio e cervical dos canais radiculares. A morfologia superficial foi avaliada em discos de dentina por meio de microscopia eletrônica de varredura (MEV). Testes de Mann-Whitney e Friedman foram utilizados para a análise estatística (α = 5%). O BCS apresentou menores porcentagens de *gaps* (p=0.00) e de espaços vazios totais (*voids* + *gaps*, p=0.01) em comparação com o BRR. Embora o BRR tenha apresentado a menor porcentagem de *gaps* no terço cervical (p=0.00), o BCS apresentou desempenho consistente em todos os terços radiculares (p>0.05). O MEV revelou que o BCS possui uma estrutura homogênea e compacta, enquanto o BRR exibe uma superfície mais porosa, com maior número de *voids* predominantemente de tamanho micrométrico ou maior. O BCS demonstrou maior capacidade de obturação em canais radiculares ovais, em comparação ao BRR. O grupo BRR apresentou a maior porcentagem de falhas nos segmentos médio e apical.

**Figure:**
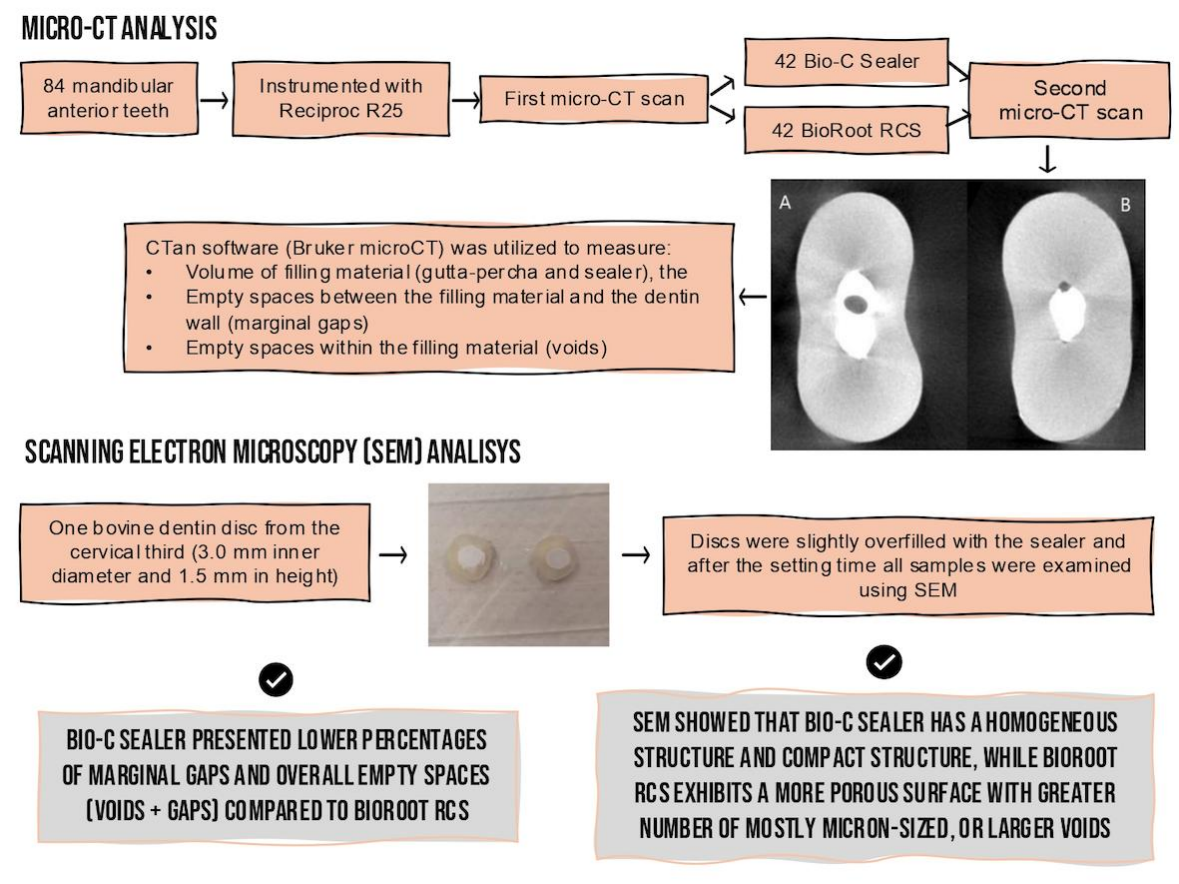

## Introduction

The main goal of root canal filling is to hermetically seal the canal space previously occupied by the dental pulp with appropriate materials after cleaning and shaping procedures^1^. The three-dimensional (3D) seal of the root canal system (RCS) is crucial to prevent the growth and migration of bacteria and their byproducts into the periradicular tissues^2^. The quality of root canal filling in terms of apical limit, homogeneity, and compaction strongly influences the long-term outcome of endodontic treatment^3^. Nonetheless, achieving a hermetic root filling remains challenging, especially due to the complex root canal morphology^4^ ^,^ ^5^. Isthmuses, pronounced curvatures, and oval root canals are anatomical features that can lead to empty spaces in the filling mass (voids) and in the interface between the gutta-percha and the canal walls (marginal gaps), which are detrimental factors that can compromise treatment longevity^5^. For a long time, the methods used for evaluating the quality of root canal fillings consisted predominantly of two-dimensional approaches or required the destruction of the sample, limiting the depth of analysis^5^. In contrast, micro-CT offers a non-destructive approach and preserves the sample for future analysis or repeated evaluations, providing 3D visualization^4^.

Oval-shaped canals, identified by their largest diameter being at least twice as large as their smallest diameter, occur in approximately 25% of teeth^6^. This root canal geometry poses significant challenges in preparation and filling procedures. Gutta-percha, the main core material commonly used in root canal filling, has no flow capacity to adequately reach small spaces within the RCS^7^. Consequently, this often results in larger sections of the canal being filled only with sealer, increasing the likelihood of voids within the filling mass^5^ ^,^ ^7^. In this context, the sealer's flowability allows it to fill the spaces left by gutta-percha, enhancing the bond strength between the filling material and the root canal walls^8^. Recently, calcium silicate-based sealers have gained increasing popularity within the endodontic community^9^. Their alkaline pH and the ability to release calcium ions have earned these materials recognition for their biocompatibility, antimicrobial properties, and bioactive potential^9^.

Calcium silicate-based sealers are commercially available in both traditional powder and liquid formulations as well as in a "ready-to-use" version. The latter is provided in a syringe, with the sealer already pre-mixed, simplifying the application process and eliminating the need for manual mixing. BioRoot RCS (BRR; Septodont, Saint-Maur-des-Fosses, France) is a calcium silicate-based sealer commercially available in a powder-liquid form since 2015. Its powder consists of tricalcium silicate, povidone, and zirconium oxide, while the liquid is an aqueous solution of calcium chloride and polycarboxylate^10^. Introduced into the market in 2018, Bio-C Sealer (BCS; Angelus, Londrina, PR, Brazil) is another calcium silicate-based sealer, provided by the manufacturer in a "pre-mixed" or "ready-to-use" syringe. Known for its capability to alkalinize the environment, BCS also features a short setting time, suitable radiopacity, and minimal volumetric change^11^. BCS exhibits a superior flow rate when compared to the "gold-standard" AH Plus (Dentsply Sirona, Tulsa, OK, USA), enhancing its capacity to navigate the complexities of the RCS^11^.

To date, there are limited studies that have explored the difference between the presence of voids and marginal gaps through micro-CT^5^, as well as the influence of the composition/formulation of calcium silicate-based sealers on the occurrence of empty spaces in oval-shaped root canals. Thus, the aim of this in vitro study was to investigate the percentage of voids and marginal gaps in oval root canals filled with two calcium silicate-based sealers, one pre-mixed (BCS) and the other in powder-liquid formulation (BRR), using the single-cone technique. The null hypotheses stated that there would be no difference in the percentage of voids and gaps: (i) between the two sealers across all canal thirds, and (ii) among the anatomical sections of the canal (coronal, middle, and apical thirds).

## Materials and methods

### Specimens preparation

The specimen’s calculation was based on data from a prior study^5^, which compared the percentage of voids and gaps when using different endodontic sealers and filling techniques, considering the following parameters: power 0.80; alpha error 0.05; effect size 0.63; for two groups (G*Power v3.1, Heinrich-Heine-Universität, Düsseldorf, Germany). As indicated by the calculation, eighty-four mandibular anterior teeth (n=42 for each sealer) were used, and for that, this study's protocol was approved by the local ethics committee (number 25436619.0.0000.5346).

For the initial sample selection, the teeth were affixed to Styrofoam supports (MOR, Santa Cruz do Sul, Rio Grande do Sul, Brazil) and placed on the i-CAT device platform (Imaging Sciences International Inc, Hatfield, PA; 120 kVp, 3-8 mA, and 0.2-mm voxel). Subsequently, the tomographic images were exported in DICOM format and analyzed using CS 3D Imaging software (Carestream Health, Rochester, NY). Brightness, contrast, and zoom settings were available. The criteria described by Girelli et al. ^5^ were utilized to select oval-shaped canals. The exclusion criteria from the tomography analysis included root fractures, the presence of cracks, internal or external root resorption, prior endodontic treatment, curvature angles exceeding 22º, roots with immature apices, and the presence of more than one root canal.

### Root canal preparation

The teeth were decoronated using a diamond disc (Komet, Santo André, SP, Brazil) close to the cemento-enamel junction. The working length (WL) was established by inserting a size #10 K-file (Dentsply Maillefer, Tulsa, OK, USA) into the canal until it was visible at the apical foramen, then 1 mm was subtracted from this length. All canals were instrumented by a single operator (I.M.L) with reciprocating nickel-titanium (NiTi) instruments (Reciproc R25; VDW, Munich, Germany) mounted on the VDW Silver motor (VDW, Munich, Germany), with the preset program ‘‘RECIPROC ALL’’. The instruments were used with a pecking motion and an amplitude of 3 mm, which progressively led to the WL. After three pecking movements, the instrument was removed from the root canal and cleaned with sterile gauze. During the procedures, the patency was maintained using a #10 K-file. The canals were irrigated with a total volume of 10 ml of 2.5% sodium hypochlorite solution (NaOCl; Asfer Indústria Química, São Caetano, SP, Brazil) using a plastic syringe and 30G needle (NaviTip, Ultradent, South Jordan, UT, USA). The EasyClean file, coupled to a counter-angle and operated with a micromotor at approximately 20,000 rotations per minute (KaVo Kerr Group, Charlotte, NC, was utilized for the final irrigation. The activation of the irrigating solutions was performed with three cycles of 20s, positioning the file up to 2 mm short of the WL, using the following sequence: 2 mL of 2.5% NaOCl, 5mL of 17% EDTA, and 2 mL of 2.5% NaOCl. A final rinse with 5 mL of saline solution (0.9% NaCl) was performed to ensure the complete removal of NaOCl and EDTA ^12^. The root canals were dried using capillary tips (Ultradent, South Jourdan, UT, USA) associated with only one sterile paper point (R25) ^13^ ^,^ ^14^.

After root canal preparation, the roots underwent a first micro-CT scan (micro-CT #1) using the inspeXio SMX-90CT Plus X-ray microtomography scanner (Shimadzu Benchtop Microfocus X-ray CT System; Shimadzu Corp., Kyoto, Japan), set at a voltage of 70 kV, amperage of 90 µA, and completing a 360º rotation around the vertical axis. The scan sequences were reconstructed with the inspeXio SMX-90CT software (Shimadzu Corp., Kyoto, Japan), utilizing a 0.01 mm voxel size to produce images with a resolution of 1024x1024 pixels and a slice thickness of 0.018 mm. The images were evaluated in DICOM format to calculate the root canal volume after instrumentation. For this evaluation, the canal was segmented based on differences in grayscale levels, and the volume of the root canal (mm³) was determined using the CTan software (Bruker microCT, Kontich, Belgium).

To ensure homogeneity between groups, the initial volume of the instrumented root canal was the morphological parameter considered for random stratification into two groups (n=42), according to the used sealers: Bio-C Sealer (BCS) or BioRoot RCS (BRR). The roots distribution was performed using a computer algorithm (random.org). The homogeneity of variances between groups, assessed by Levene’s test, confirmed no differences (p=0.283) in the initial root canal volumes across both groups.

### Root canal filling

Prior to the root filling procedures, Reciproc R25 gutta-percha cones (VDW, Munich, Germany) were selected, and their radiographic fit was verified to ensure tight apical binding and proper adaptation 1 mm short of the apex. The sealers were used in accordance with the manufacturer’s instructions and proportions. All the roots were filled using the single cone technique, as follows:

BCS was applied using a syringe and plastic needles provided by the manufacturer. The pre-mixed sealer was injected into the root canal close to the WL with the plastic needle provided by its manufacturer. While for BRR, the sealer was mixed according to the manufacturer's instructions: using the spoon provided by the manufacturer, a portion of powder was placed on a glass plate and five drops of liquid were added. During spatulation, the powder was progressively added to the liquid until the mixture formed a smooth paste, a process that took approximately 60 seconds. Afterward, the same protocol was followed for both sealers: a #25 lentulo spiral (Dentsply Sirona), attached to a low-speed motor, and a #20 K-file was employed in pumping motions to distribute the sealer evenly along the canal walls. Lastly, the gutta-percha cone was carefully inserted into the canal until it reached the WL ^4^ ^,^ ^15^.

Digital radiographs in the buccolingual direction were captured in all specimens to confirm the homogeneity of the filling. For all specimens, excess gutta-percha in the coronal portion was cut using a heated plugger (SSWhite Duflex, Juiz de Fora, MG, Brazil). Subsequently, a cold plugger was used to lightly compact the material. The cervical portion of the canals was sealed with a temporary restorative material (Cavit G; 3M-ESPE, Seefeld, Germany) and then stored at 37ºC with 95% relative humidity for 2 weeks to ensure the sealers were fully set. The specimens were subjected to a new scan (micro-CT #2) to determine the total volume of the filling material, using the same scanning parameters previously described.

### Micro-CT analysis

**Figure 1:**
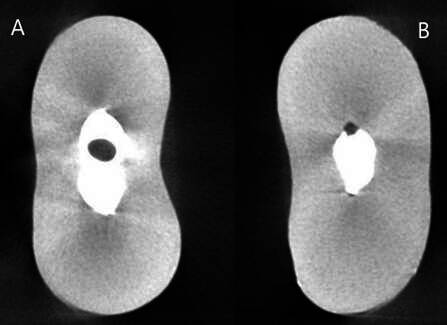
Representative micro-CT images of the cross sections of the root canal filling. **(A)** Empty space inside the filling material (void). **(B)** Empty space between the filling material and the dentin wall (marginal gaps).

The images obtained from micro-CT #2 were reconstructed following the procedures described for micro-CT #1. The volume of marginal gaps and voids within the filling mass were calculated according to Girelli et al. (2023) ^5^ with minimal adaptations. The CTan software (Bruker microCT) was utilized to measure the volume of filling material (gutta-percha and sealer), the empty spaces between the filling material and the dentin wall (marginal gaps), and the spaces within the filling material (voids), as shown in Figure 1. All analyses were performed by a trained examiner who was blinded to the allocation groups. Grayscale intervals for identifying each study object were determined using a density histogram through adaptive thresholding. To obtain the percentage of voids (V_void%_) and gaps (V_gap%_), the following formulas were used ^5^, respectively: V_void%_ = V_void_ *100 / V_filling_; V_gap%_ = V_gap_ *100 / V_filling_. Analysis was conducted for the complete canal and separately by root third. Lateral or accessory canals were not considered in the analysis.

### Scanning electron microscopy

As a qualitative and complementary analysis, the surface morphology of each sealer was evaluated by SEM. For each material, one bovine dentin disc from the cervical third (3.0 mm inner diameter and 1.5 mm in height) was obtained with the aid of a precision cutting machine (Isomet; Extec Corp, Enfield, CT, USA) set at 300 rpm and equipped with a double-sided diamond disc (Buehler, Lake Bluff, IL, USA). The disc was placed on a flat glass plate, which was covered with a polyethylene plastic sheet. The discs were slightly overfilled with the sealer, and another glass plate (also covered with a plastic sheet) was pressed on top to create a flat and uniform sealer surface ^16^. The samples were then incubated for 24 hours at 37°C and 95% relative humidity. After incubation, all samples were coated with a layer of gold and subsequently examined using SEM (EVO LS-15; Zeiss, Oberkochen, Germany), with 20 kV, at 1,320× and 10,200× magnifications. SEM findings were analyzed descriptively.

### Statistical analysis

After submitting data to normality tests, nonparametric statistics were used to assess differences between groups. Mann-Whitney test was applied to compare both groups of sealers in each root third and in total. Friedman and post-hoc tests were used to compare the root thirds in each group. The significance level was set at p < 0.05, and all the analyses were performed using the SPSS software (version 21.0, SPSS IBM, Armonk, NY, USA).

## Results

The mean and standard deviation for the volume of the root canal filling materials and the percentage volume of voids and marginal gaps are described in Table 1. The mean filling of the entire root canal was 5.85 ± 1.84 mm³ for BCS and 5.54 ± 1.64 mm³ for BRR (p=0.46). Regarding the overall empty spaces (voids + gaps), roots filled with BCS showed a lower percentage of empty spaces compared to those filled with BRR (BSC 0.14 ± 0.16; BRR=0.25 ± 0.25; p=0.01). The same trend was observed in the presence of marginal gaps; BCS presented a significantly lower percentage of marginal gaps than BRR (BCS=1.86 ± 2.67; BRR=3.31 ± 3.39; p=0.00). Regarding the presence of voids, no differences were found between the two sealers (p=0.19).

In the comparison of the sealers across each root third, BRR exhibited a significantly greater percentage of marginal gaps compared to BCS in both the middle (BSC=2.54 ± 5.27; BRR=5.05 ± 7.4; p=0.00) and apical thirds (BSC=1.82 ± 3.56; BRR=7.50 ± 10.42; p=0.00). Similarly, for the percentage of voids, a notable difference was observed in the apical third, with BRR showing higher values than BCS (BCS=0.63 ± 1.73; BRR=3.48 ± 14.91; p=0.00).

**Figure 2:**
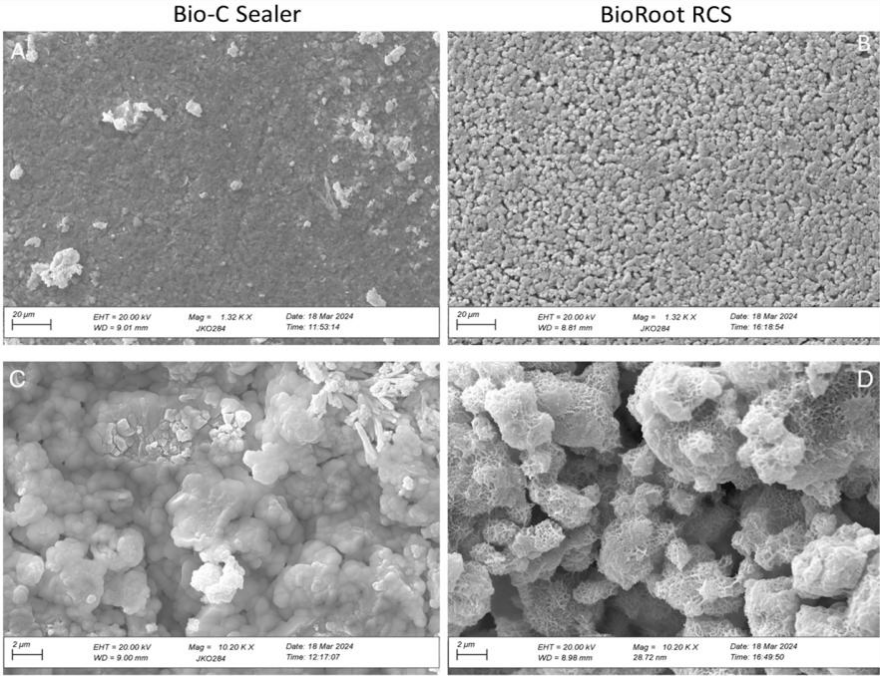
Representative 3D images of voids and marginal gaps after root canal filling with Bio-C Sealer (A) or BioRoot RCS (B).

Within the BRR group, the cervical root third had the lowest percentage of marginal gaps compared to middle and apical thirds (cervical=1.17 ± 1.96; middle=5.05 ± 7.45; apical=7.50 ± 10.42; p=0.00). On the other hand, BCS showed similar V_gap%_ in all the root thirds (p>0.05). Regarding the percentage of voids, no differences were found among the thirds for none of the sealers (p=0.29 for BCS; p=0.18 for BRR). Figure 2 shows representative 3D images of the marginal gaps and voids from each group after root canal filling.

SEM analysis revealed that on the millimeter scale, BCS is homogeneous, but BRR is not. The BRR bovine disc consists of 0.3‒0.5 mm crystal laths that sit in a "groundmass" of orders of magnitude smaller grains. On the micro-scale, BCS has a compact structure with only a few, mostly sub-micrometer voids (Figure 2a). The 'groundmass' of BRR is more porous, with a greater number of mostly micron-sized, or larger voids (Figure 2b). On the nanoscale, individual micron-sized globules making up BCS are in direct contact (Figure 2c), with only a few gaps in between. In contrast, the micron to few-micron diameter particles of BRR "groundmass" are connected to each other by columnar and lamellar "bridges", which adds further porosity (Figure 2d).

**Table 1:** Filling volume (mm^3^) and percentage of voids and gaps (Mean ± standard deviation) in the different thirds and in the whole canal after root canal filling.

Measurement		Bio-C Sealer	BioRoot RCS
		Mean ± SD	Mean ± SD
Filling volume (mm^3^)	Cervical	3.01 ± 1.02^A^	2.98 ± 0.99^A^
	Middle	1.98 ± 0.65^A^	1.86 ± 0.54^A^
	Apical	0.86 ± 0.25^A^	0.75 ± 0.20^A^
	Total	5.85 ± 1.84^A^	5.54 ± 1.64 ^A^
Marginal gaps (%)	Cervical	1.34 ± 2.29^Aa^	1.17 ± 1.96^Ab^
	Middle	2.54 ± 5.27^Ba^	5.05 ± 7.45^Aa^
	Apical	1.82 ± 3.56^Ba^	7.50 ± 10.42^Aa^
	Total	1.86 ± 2.67^B^	3.31 ± 3.39^A^
Voids (%)	Cervical	0.51 ± 1.1^Aa^	0.91 ± 3.23^Aa^
	Middle	0.60 ±1.60^Aa^	0.77 ± 1.99^Aa^
	Apical	0.63 ± 1.73^Ba^	3.48 ± 14.91^Aa^
	Total	0.59 ± 1.18^A^	1.30 ± 2.89^A^
Overall empty spaces (Voids + gaps)		0.14 ± 0.16^B^	0.25 ± 0.25^A^

Different uppercase letters indicate significant differences in each row (Mann-Whitney test, p<0.005). Different lowercase letters indicate significant differences in each column (Friedman test, p<0.005).

## Discussion

The presence of voids and gaps in the root canal filling is of critical concern, as it may be linked to long-term treatment failures by compromising the hermetic seal of the canal space ^17^. Therefore, the present study aimed to investigate the percentage of voids and marginal gaps in oval root canals filled with two calcium silicate-based sealers, one pre-mixed (BSC) and the other in powder-liquid formulation (BRR), using the single-cone technique.

In the present study, neither BCS nor BRR provided void-free root canal filling using the single-cone technique, which is consistent with findings from previous research ^1^ ^,^ ^5^ ^,^ ^15^. This recurring observation highlights the challenges of achieving complete root canal obturation, especially in oval-shaped canals where the buccolingual diameter significantly exceeds the mesiodistal diameter ^6^. This geometrical shape poses considerable obstacles for instrumentation and obturation, as evidenced by the existing literature ^6^ ^,^ ^15^ ^,^ ^18^ and confirmed by our results. Moreover, the centric positioning typically achieved by NiTi instruments during chemomechanical preparation often leaves a considerable portion of the canal's oval areas uninstrumented, further contributing to the formation of voids and gaps within the filled canal ^19^.

In this study, we opted for the single-cone method because it is simple, cost-effective, and easy to reproduce ^20^. Nonetheless, one may argue that the use of the single cone technique is not the best technique for the obturation of oval-shaped canals. However, the decision for the single-cone technique was further influenced by the compatibility of this technique with calcium silicate-based sealers. Such sealers are specifically recommended for the single-cone approach because the application of heat, as used in other techniques, could detrimentally alter their characteristics, including viscosity, bond strength, and setting time ^20^ ^,^ ^21^. To minimize the formation of voids and gaps, a Lentulo spiral was used to distribute the sealer along the root canal walls before inserting the master gutta-percha cone. According to the literature, utilizing a Lentulo spiral or a K-file prior to the placement of the gutta-percha cone significantly reduces the occurrence of voids ^22^ ^,^ ^23^.

The first null hypothesis tested in this study was rejected since the observed data reveal that BCS promoted a lower percentage of overall empty spaces and marginal gaps compared to BRR (Table 1). The high flow level of BCS may account for its effective filling performance in oval areas, as suggested by Nomura et al. ^24^. Previous research findings also demonstrate a higher percentage of empty spaces in roots filled with BRR, when compared to epoxy resin-based sealers and to other calcium silicate-based or bioactive glass sealers ^25^ ^,^ ^26^ ^,^ ^27^. As a powder-liquid sealer that requires manual mixing, BRR may have incorporated air into the material during mixing, leading to void formation even before obturation ^27^. Furthermore, the quality of the mix is highly dependent on the clinician's spatulation technique, which can significantly affect the overall quality of the mixture.

In the present study, roots filled with BRR showed a higher percentage of voids and marginal gaps, particularly in the apical and middle thirds. These findings are consistent with the SEM images (Figure 3), which revealed greater inhomogeneity and a more porous structure in the BRR sealer compared to BCS. The BRR sealer showed a more porous and inhomogeneous microstructure, with irregular crystal aggregates and numerous micron-sized voids. Such surface morphology likely promotes air entrapment during placement and reduces adaptation to dentinal walls, explaining the greater incidence of empty spaces detected by micro-CT. Conversely, the homogeneous and compact structure of BCS observed under SEM suggests improved flow and interfacial adaptation, which corroborates the lower percentages of gaps and voids measured in the micro-CT analysis.

Regarding voids, similar total percentages were found for BCS and BRR sealers. However, in the apical third, BCS showed better filling performance. Most prior studies have assessed the presence of empty spaces without distinguishing between voids and marginal gaps ^4^ ^,^ ^15^ ^,^ ^26^ ^,^ ^28^. However, with the use of micro-CT, this study differentiated voids as the empty spaces within the filling mass and gaps as the empty spaces between the filling material and the root canal walls. Although the definitive relationship between the presence of voids and endodontic treatment outcome has not been firmly established ^29^, it is conjectured that voids in root fillings might be deemed less critical clinically since they encase bacteria in a hostile setting. Nonetheless, gaps between the filling material and the dentinal walls present a potentially higher risk. These empty spaces, being in contact with potentially contaminated canal walls, could compromise the integrity of the seal and lead to leakage, posing a threat to treatment success ^29^ ^,^ ^30^.

The second null hypothesis was partially rejected, as significant differences in gap presence were observed among the different root thirds for the BRR group. Specifically, the cervical third displayed the lowest percentage of gaps. Furthermore, although the difference is not statistically significant for BCS, the apical third exhibited the highest percentage of voids for both sealers. Minimizing empty spaces in the apical third is crucial, given its critical role in the success of endodontic treatments ^31^. Gutta-percha is frequently employed alongside sealers to ensure a fluid-tight seal and to minimize the thickness of the sealer layer ^7^ ^,^ ^30^. Manufacturers provide single-file instrumentation systems that include corresponding gutta-percha cones, with the aim of making root canal obturation more precise. However, despite efforts towards standardization, in the Reciproc system, significant differences between the file and the gutta-percha cone were observed, with the cone demonstrating smaller diameters and tapers compared to the file ^32^. It seems possible that this mismatch contributed to the increased formation of voids.

Mandibular anterior teeth were selected for this study due to their root canal morphology, characterized by the presence of long oval-shaped canals in over 50% of these teeth ^6^. To ensure a homogeneous sample, 3D imaging was used for initial sample selection, focusing on pre-operative morphological characteristics. Furthermore, teeth were allocated using stratified randomization, considering the root canal volume following preparation. The assessment of voids within root canal fillings is not a novel concept. Historically, various methods such as radiography, root cross-sectioning, and dye injection followed by diaphanization have been utilized for this analysis ^33^ ^,^ ^34^. However, in addition to lacking precision, some of these methods require sample destruction. In contrast, micro-CT stands out as a non-destructive method, well recognized for its ability to perform high-definition 3D analyses ^5^ ^,^ ^7^ ^,^ ^26^.

It is important to point out that in micro-CT images, artifacts generated by the obturation materials can mask these spaces, potentially leading to underestimation. Similar to a previous study that used micro-CT as a gold-standard reference for volumetric ^35^ analysis, our protocol employed high-energy settings and adaptive thresholding to minimize the impact of X-ray artifacts. According to Celikten et al. (2018) ^35^, resin-based sealers such as AH Plus produce more blooming artifacts than calcium silicate-based sealers due to the presence of heavy radiopacifies like calcium tungstate and zirconium oxide. In contrast, bioceramic sealers with lower-density radiopacifiers generate fewer X-ray artifacts and provide better image accuracy. This supports the present findings, as both sealers evaluated here are calcium silicate-based materials expected to produce fewer radiographic distortions.

Root canal treatment produces an irregular physical smear layer and dentin debris, which interfere with disinfection procedures and limit the penetration of irrigants, medicaments, and filling materials ^36^. This challenge becomes even more critical in teeth with oval-shaped canals, where the anatomical complexity hinders the complete removal of debris and smear layer, particularly from untouched canal recesses ^37^ ^,^ ^38^. In the present study, the final irrigation was performed with the aid of the EasyClean file to enhance the removal of residual hard-tissue debris and promote more effective cleaning of the root canal system. A previous study demonstrated that EasyClean is capable of efficiently removing debris, showing comparable performance to well-established agitation techniques such as EndoActivator ^39^.

This study has limitations inherent to in vitro methodologies. Also, some may question the use of the R25 instrument for root canal preparation. However, the choice was based on the morphological characteristics of the selected samples and aimed to preserve dentin structure ^40^. Nevertheless, we acknowledge that this decision could have influenced the obturation quality observed in the apical third. Future studies comparing different instrument sizes and their impact on the adaptation of calcium silicate-based sealers would be valuable to clarify this aspect. In addition, all micro-CT analyses were performed by a single trained examiner, which may introduce operator-related bias. Although this approach follows the methodology adopted in several studies ^4^ ^,^ ^5^ ^,^ ^26^, the inclusion of multiple blinded evaluators could further strengthen data reliability.

## Conclusion

It could be concluded that the BCS offers superior filling performance in oval-shaped canals compared to BRR. In terms of root canal thirds, the middle and apical sections were found to have a greater percentage of gaps compared to the cervical third in the BBR group. Further studies are recommended to improve the sealing effectiveness of calcium silicate-based sealers, aiming to enhance the success of endodontic treatments.
